# Novel neurosteroid therapeutics for post-partum depression: perspectives on clinical trials, program development, active research, and future directions

**DOI:** 10.1038/s41386-023-01721-1

**Published:** 2023-09-15

**Authors:** Riah Patterson, Irina Balan, A. Leslie Morrow, Samantha Meltzer-Brody

**Affiliations:** 1 Department of Psychiatry and Emergency Medicine, MacNider Bldg. Suite 304, CB# 7160, Chapel Hill, NC 27599-7160 USA; 2 Department of Psychiatry, Bowles Center for Alcohol Studies, 3027 Thurston Bowles Building, CB 7178, Chapel Hill, NC 27599-7178 USA; 3 Department of Psychiatry and Pharmacology, Bowles Center for Alcohol Studies, 3027 Thurston Bowles Building, CB 7178, Chapel Hill, NC 27599-7178 USA; 4 Department of Psychiatry, UNC Center for Women’s Mood Disorders, MacNider Bldg. Suite 304CB #7160, Chapel Hill, NC 27599-7160 USA

**Keywords:** Drug development, Neuroimmunology

## Abstract

This article reviews novel neurosteroid therapeutics for post-partum depression, with a focus on their development, clinical trial data, current practices, and future directions in this exciting field. We discuss the clinical impact of brexanolone and several other neurosteroids, particularly as they relate to the treatment of postpartum depression (PPD) and major depressive disorders outside of the perinatal period. There has been increasing interest in GABA signaling and modulation as it pertains to the development of altered circuity and depressive states. This scientific underpinning served as the rationale for the initial development of brexanolone. We review the clinical trials supporting its Food and Drug Administration (FDA) approval as the first rapidly acting antidepressant specific for PPD, and the subsequent development of a clinical brexanolone program at an academic medical center, highlighting new research and data from that site as well as the challenges with the delivery of this I.V. drug. In addition to the GABA signaling hypothesis, we discuss the new evidence demonstrating that brexanolone inhibits inflammatory signaling post-infusion, suggesting that inflammatory signaling may contribute to the etiology of PPD. Finally, we describe new and future directions in neurosteroid therapeutics, including the development of an oral agent, zuranolone, and the IV and oral formulations of ganaxolone. Ultimately, the hope is that these novel neurosteroid therapeutics will provide fast-acting treatment for these impairing disorders and improve our understanding of the underlying mechanisms of depressive disorders.

## Introduction: underlying pathophysiology of postpartum depression

Postpartum depression (PPD) is one of the most common complications of the perinatal period and has significant maternal morbidity and adverse consequences for the mother, infant and family [1]. Maternal suicide is one of the greatest causes of maternal mortality. The perinatal period a time of significant fluctuations in the reproductive hormones (steroids), estrogen and progesterone. Over the third trimester of pregnancy the levels of estrogen and progesterone markedly increase (from 10 fold to 50 fold compared with normal levels outside of pregnancy), and are accompanied by activation of the hypothalamic-pituitary-adrenal (HPA) axis and consequently, greatly increased levels of cortisol. At the time of childbirth and following delivery of the placenta, the levels of estrogen and progesterone rapidly decrease and the HPA axis must quickly compensate in response to these dramatic hormonal fluctuations [2, 3].

The reproductive steroids also have a powerful role in regulating neural function and have been shown to have significant impact on the pathophysiology of depression, particularly in women [4]. Specifically, reproductive steroids regulate synthetic and metabolic enzymes of neurotransmitters including dopamine, norepinephrine, serotonin, glutamate, and gamma-aminobutyric acid (GABA). In addition, the reproductive steroids play a prominent role in regulation of stress and the HPA axis at multiple levels including the hypothalamic corticotropin releasing hormone (CRH) gene and multiple interactions between the estrogen and glucocorticoid receptors [5, 6].

The gold standard pharmacologic treatment for PPD has been focused on using antidepressants indicated for major depressive disorder outside of the perinatal period, with the selective serotonin reuptake inhibitors (SSRIs) being the most common. However, the SSRIs or other antidepressants do not offer a rapid treatment response, nor are they specific for PPD. Further, many women do not have an adequate response to treatment and do not achieve remission of symptoms [7]. Consequently, there has been a great unmet need for novel therapies for PPD that are both specific and address the underlying pathophysiology. Further, there has been a strong need to develop antidepressant therapies that are fast acting and can relieve the suffering associated with a depressive episode as quickly as possible. This is particularly important during the postpartum period, which is a uniquely vulnerable time for the mother, baby, and family. During the acute postpartum period, the mother (and family) has substantial caretaking responsibilities of a new baby, and it is a critical window for mother-infant attachment. In mothers with postpartum depression, there can be disrupted parenting that may manifests as decreased maternal responsiveness, disengagement, withdrawal or intrusiveness to the infant [8, 9]. Consequently, there is an urgent need for rapidly acting treatment to address the burden of symptoms associated with postpartum depression.

In this paper, we review the literature to date on novel neurosteroid development in postpartum depression including clinical trials, program development, active research, and future directions in this important area, which has significant public health implications across two generations.

## Neurosteroid and GABAergic signaling hypothesis

There are a number of hypotheses of the underlying pathophysiology of PPD including differential sensitivity to gonadal hormonal fluctuations during pregnancy and the postpartum transition, dysregulation of the HPA axis, changes in synaptic transmission of GABA, altered levels of immune system factors, and altered neural network activity [10–14].

The female reproductive hormones play important roles in the regulation of most neurotransmitters, i.e., serotonin, norepinephrine, dopamine, GABA, and glutamate. They also have a significant function in the regulation of the HPA axis and stress reactivity [15]. The GABAergic signaling hypothesis in PPD has been of great interest for some time. Preclinical and clinical studies in PPD have demonstrated the potential roles of dysfunctional GABAergic signaling, and specifically, allopregnanolone levels, in the development of PPD. Allopregnanolone, the neuroactive metabolite of progesterone, is a powerful modulator of GABA_A_ receptors, the primary inhibitory receptor in brain. Stress reactivity is also associated with GABA signaling impairment. There are two potential contributors of the observed impairment. First, alterations in GABA signaling can be due to fluctuations in reproductive hormone levels in those who are differentially sensitive, likely due to underlying genetic vulnerability. This point was first shown by the work of Maguire and Mody (2008), who investigated mouse models of PPD, that were bred to have genetically-induced disturbances of GABA_A_ receptors [6]. The female mice showed normal behaviors until delivery, when a change occurred and the mice then demonstrated symptoms of stress, depression and failed to exhibit maternal behaviors, that were associated with hyper-activation in the prefrontal cortex and amygdala [6]. The observation that these behavioral changes in the mice were all reversed by the administration of allopregnanolone served as compelling evidence for the development of brexanolone, a proprietary formulation of allopregnanolone. The second contributor is that endogenous allopregnanolone levels drop precipitously in brain at parturition [16] suggesting that the rapid loss of circulating allopregnanolone may also contribute to the syndrome in vulnerable women. Brexanolone became the first Food and Drug Administration (FDA) approved treatment for PPD in human mothers, which is administered intravenously over 60 hours and has demonstrated rapid and sustained improvement in symptoms of PPD [17].

## Clinical trial development of brexanolone

The development of brexanolone from the initial open-label trial to FDA approval occurred over 4 years. The initial protocol for an open-label proof of concept study enrolled the first patient in January of 2015 and the drug was approved by the FDA in March of 2019 after phase 2 and 3 positive clinical trials. The first open-label patient was recruited from the University of North Carolina at Chapel Hill Perinatal Psychiatry Inpatient Unit (UNC’s PPIU) for participation in the trial. The inclusion and exclusion criteria developed for the open-label trial remained largely identical over all phases of clinical trial development. Specifically, postpartum mothers were eligible for participation if they had a formal diagnosis of a major depressive episode starting no earlier than the third trimester and no later than 12 weeks after childbirth. Severity of depression was assessed by the 17-item Hamilton Depression Rating Score with scores of ≥20 (HAM-D; [18]) required for inclusion. If eligible patients were on antidepressant medication for more than 2 weeks at a stable dose, they were permitted to continue the antidepressant over the course of the 60 hour brexanolone infusion period.

The infusion schedule of brexanolone was chosen to replicate third-trimester levels of allopregnanolone (~150 nM, [19]). The protocol was designed as a 60 hour infusion, beginning with a 12-hour dose titration on Day 1 in which brexanolone was infused at 25%, 50%, then 75% of the maintenance dose for 4 hours at each level. The maintenance dosing began at Hour 12 and was administered for 36 hours to reach the steady‐state plasma concentration of ~150 nM. The tapering of the dose began at Hour 48, was then decreased over the next 12 hours to 75%, 50%, and then to 25% of the maintenance with the goal of enhancing physiologic adjustment to the reduction of allopregnanolone levels.

The protocol used during the open-label study was adjusted such that in phase two and three clinical trials subjects received brexanolone at 30 µg/kg per h (0–4 h); 60 µg/kg per h (4–24 h); 90 µg/kg per h (24–52 h); 60 µg/kg per h (52–56 h); 30 µg/kg per h (56–60 h). Despite this slight protocol change, all study participants had similar inclusion and exclusion criteria and were within 6 months of childbirth. The phase 2 study was a double-blind placebo-controlled study of a single, continuous 60 hour infusion of brexanolone or placebo was conducted between December 2015 and May 2016 and enrolled 21 women overall (*N* = 10 assigned to brexanolone and *N* = 11 to placebo). The primary study endpoint after 60 hour of infusion was the change from baseline in the 17-item HAM-D total score. All patients who completed the infusion were followed out to 30 days.

After 60 hours of infusion in the phase two study, the mean change (reduction) in HAM-D total score from baseline was 21 points in the study patients treated with brexanolone compared to 8.8 points in the placebo group (difference −12·2, 95% *p* = 0·0075; effect size 1.2). No deaths, serious adverse events, or discontinuations because of adverse events were reported in either group [20]. The positive results from the phase 2 study provided the rationale to move to phase 3.

There were a total of three positive randomized, double-blind, placebo-controlled trials of brexanolone versus placebo that demonstrated a sustained response to brexanolone infusion through 30 days of follow-up post treatment [17]. Brexanolone infusion showed statistically significant reductions in HAM-D scores as compared to placebo, both with or without baseline antidepressant medication. Participants were enrolled in the phase 3 trials between during 2016–2017. There were 375 women screened simultaneously across both studies. In study 1, 138 were randomly assigned to receive either brexanolone 90 μg/kg per h (*n* = 45), or brexanolone 60 μg/kg per h (*n* = 47), or placebo (*n* = 46). In study 1, after the 60 hour brexanolone infusion, the least-squares (LS) mean reduction in HAM-D total score from baseline was 19.5 points in the brexanolone 60 group and 17.7 points in the brexanolone 90 μg/kg per hr group compared with 14.0 points in the placebo group (difference −5·5 [*p* = 0·0013) for the brexanolone 60 μg/kg per hour group; −3·7 [95% CI −6·9 to −0·5], *p* = 0·0252 for the brexanolone 90 μg/kg per hr group). In study 2, 108 were randomly assigned to receive brexanolone 90 (*n* = 54) or placebo (*n* = 54). After the 60 hour infusion, the LS mean reduction in HAM-D total score from baseline was 14.6 points in the brexanolone 90 μg/kg per h group compared with 12.1 points for the placebo group (difference −2·5 [95% CI −4·5 to −0·5], *p* = 0·0160) [17]. It is also important to note that the placebo response was robust in the phase 3 studies, which is consistent with past clinical trials for major depressive disorder [21, 22]. Nonetheless, the studies demonstrated statistically significant and clinically meaningful treatment differences between active drug (brexanolone) and placebo groups. Given the rapid onset of action and efficacy demonstrated in all of the clinical trials, brexanolone was demonstrated to be a powerful tool for treatment of patients with severe PPD [17].

Throughout all phases of the clinical trials, the drug was well-tolerated overall. The most commons side effects included flushing or hot flashes, dry mouth, and sleepiness. Sedation and in rare instances, a brief loss of consciousness were reported. All episodes of sedation fully resolved within 90 minutes during clinical trials when the dose was reduced. The FDA approved brexanolone in March of 2019 with the requirement of a Risk Evaluation and Mitigation Strategy (REMS). REMS specifies the procedures that must be followed for brexanolone to be administered, including appropriate supervision in a medical setting which provides access to continuous pulse oximetry and sedation scale monitoring every 2 hours.

## Post-FDA approved clinical brexanolone programs: the example of UNC

The FDA-approval of medication specifically for postpartum depression was hugely exciting in the perinatal psychiatry field, as previously PPD was treated similarly to a major depressive episode with limited clinical successes. Unfortunately, most PPD cases are unrecognized, those that are recognized are undertreated, and those that are treated still rarely reach remission [7]. Thus, the availability of a new medication, with a new delivery system, and new mechanisms of action presented great treatment potential for this common and distressing illness.

In approving brexanolone, the FDA took great care to make sure these vulnerable women would be able to tolerate the drug and avoid any adverse outcomes. The REMS structure was established to ensure that brexanolone infusion would be conducted in a supervised medical setting with access to continuous pulse oximetry and frequent sedation scale monitoring. The rare episodes of excessive sedation (4–5%) were noted in the clinical trials and most commonly linked to concomitant benzodiazepine use [17]. UNC’s Clinical Brexanolone Program recommends patients discontinue, or at least reduce benzodiazepine use by 50%, prior to the infusion. There have not been any excessive sedation adverse events with this protocol in place [23].

The REMS process ensures that the medical provider, nurses, and pharmacy are all trained in safety precautions with the use of brexanolone. However, these provisions have also made delivery of brexanolone challenging. For example, during the clinical trials, brexanolone was given on UNC’s PPIU. The PPIU is a specialized 5-bed psychiatric unit caring for women who are pregnant or post-partum in need of an inpatient level of psychiatric care, but it does not have telemetry and continuous pulse oximetry that can be monitored remotely. To address these new FDA guidelines, the Clinical Brexanolone Program at UNC changed locations for the infusion site to a medical floor at UNC Hospitals. There are many benefits to being on a medical floor; the patients have more autonomy in terms of single rooms with access to their cell phones, TV, dining services, and open visitation. However, this also introduced new concerns for the safety and treatment of acute psychiatric patients. Staff on these units are not trained in perinatal psychiatry, and so there has been the need for providing additional staff education for management of psychiatric patients as well as the need for additional staff if any safety or suicidality concerns arise.

One set of challenges that continue to exist include insurance authorization and access to care. Although PPD necessitates immediate treatment, insurance providers can take up to 15 business days before they are required to respond to prior authorization requests. Prior authorization is necessary given the high cost of treatment, on average around $36,000 for the drug alone. It is also notable that this drug has been predominantly accessible to white, married women, with private insurance [23]. Women of color or with less resources, especially related to childcare, have not had equitable access to this treatment. Additional access complications include the barrier of location. Sage Therapeutics reports that they have 50 infusion sites around the country, but UNC is the only active site in North Carolina, and 3 surrounding states do not have any infusion locations. These logistical challenges make brexanolone difficult to access for many in need.

Despite some of the complications of offering brexanolone for moderate to severe post-partum depression, we have been able to offer this service continuously since brexanolone approval, and our staff report great pleasure in observing the dramatic and rapid improvement in some patients’ moods and ability to function.

## Post-FDA approved Brexanolone Research

With an established protocol in place, we have infused over 60 women with commercially available brexanolone in our clinical program at UNC since its approval in 2019. These cases came from internal referrals through our UNC Center for Women’s Mood Disorders, our PPIU and through Sage Therapeutics. In 2021, we published the first article that outlined our unique program at an academic medical center, as well as 90-day naturalistic follow up data for our first cohort of 16 patients [23]. Previously, the clinical trials only followed patient HAM-D rating scores through 30 days post-infusion. Our post-infusion data were similar to the clinical trials, with HAM-D scores dropping from 23.9 to 7.6 at the end of the 60 hour infusion, such that 94% of the cases reached a clinically meaningful reduction in HAM-D score and 56% achieved remission with a HAM-D score of 7 or below. Next, we reached 11 of our original 16 patients who provided follow up data that showed an even lower mean HAM-D score of 6.7 at 90 days post-infusion. These naturalistic data suggest that those who benefit from brexanolone will continue to benefit in the months following treatment, which is very meaningful to those suffering from PPD.

As mentioned above, there are many successes with commercial brexanolone despite the limitations that it requires a multiple day hospital admission, time away from family, and its cost. Untreated PPD can have devasting consequences for the family including poor bonding and attachment, higher rates of substance use, suicide, neglect, and poor social and emotional development of the infant [24–28]. We thus hope to better understand the mechanisms of action of brexanolone and the etiologies of PPD to allow for development of more practical and affordable bioavailable compounds for treatment of this common illness.

While the etiology of postpartum depression is believed to be multifactorial, there is increasing evidence that inflammatory signaling plays a significant role. For example, pro-inflammatory signaling is associated with major depression [29–34], and PPD [35–38]. Furthermore, proinflammatory immune activation may induce depression in healthy individuals [33] or depression-like behavior in animal models of depression [39, 40].

Laboratory studies have shown that allopregnanolone inhibits inflammatory signaling through toll-like receptors (TLRs) in mouse and human macrophages and rat brain [14, 41, 42]. TLRs are the primary site of inflammatory activation that produces cytokines and chemokines in the immune system and brain. TLRs possess the ability to detect diverse pathogenic agents, endogenous molecules, and addictive substances, which are collectively referred to as pathogen-associated molecular patterns (PAMPs), damage-associated molecular patterns (DAMPs), and xenobiotics (XAMPs) respectively. When PAMPs, DAMPs, or XAMPs are recognized by TLRs, signaling pathways are initiated, culminating in the activation of transcription factors. This activation subsequently triggers the production of inflammatory cytokines and chemokines, such as tumor necrosis factor alpha (TNF-α), interleukin-1 beta (IL)-1β, IL-6 and monocyte chemoattractant protein-1 (MCP-1) [14, 42–44]. It is worth noting that excessive TLR activation has been implicated in the pathogenesis of numerous inflammatory and neuroinflammatory diseases [29, 43, 45]. Specifically, the TLR4 signal has been implicated in a range of neuropsychiatric conditions including stress and major depressive disorder [46, 47]. The TLR7 signal has been found to enhance contextual fear memory and induce depression-like behaviors [48]. Indeed, early studies showed allopregnanolone inhibition of the inflammatory cytokines TNF-α and IL-1β in brain using an animal model of traumatic brain injury [49], while later studies showed allopregnanolone inhibition of multiple inflammatory mediators produced by the TLR 2, 4 and 7 inflammatory pathways in the alcohol preferring P rat brain [42, 44].

Over the past several years, we have investigated the potential role of the inhibition of pro-inflammatory cytokines in the therapeutic efficacy of brexanolone in studies in our PPD patients [36]. We collected whole blood prior to and after the brexanolone infusion. Whole blood cell lysates were examined for inflammatory markers within one hour and then whole blood cells were studied for subsequent in vitro responses to the inflammatory activators lipopolysaccharide (LPS) and imiquimod (IMQ). We found that brexanolone reduced baseline levels of the inflammatory markers TNF-α and IL-6 and these effects predicted HAM-D score improvement. Furthermore, brexanolone treatment inhibited the blood cell response to the inflammatory immune activators LPS and IMQ, indicating that it blocked activation of TLR4 and TLR7 that are activated by LPS and IMQ, respectively. These responses also predicted HAM-D score improvement in the patients. This is a tremendous advancement in our understanding of why brexanolone is effective and further suggests that inflammatory signaling may contribute to the etiology of PPD. Still, we do not know the duration of the inhibition of inflammatory signaling post-infusion. We do know that clinically, brexanolone is still effective at 90 days, but it is now critical to understand how long the systemic inflammatory pathways are inhibited since this inhibition may account for the long-lasting actions of brexanolone.

## Future directions

As mentioned above, our theoretical framework posits that activation of inflammatory pathways, including the production of TNF-α, IL-1β and IL-6 contributes to PPD and that inhibition of the inflammatory pathways that produce these mediators contributes to clinical remission. Allopregnanolone may achieve its remarkable and sustained therapeutic effects by inhibiting these pathways and/or by changing the sensitivity of immune cells to subsequent stimulation by inflammatory activators such as LPS. Furthermore, brexanolone infusion may impact other protective, trophic, or anti-inflammatory mediators that could also contribute to its therapeutic actions. Finally, it remains important to examine the immune responses in patients who do not respond to brexanolone therapy (typically ~ 30% of those treated [17]) to determine if baseline or post-infusion inflammatory markers or endogenous steroids differ compared to the group of patients who respond to brexanolone therapy.

These studies will provide important insights into new mechanisms of the antidepressant effects of brexanolone and may lead to development of more bioavailable medications or formulations with similar therapeutic properties. However, these studies do not negate the important role of allopregnanolone actions at GABA_A_ receptors in the therapeutic actions of brexanolone. Indeed, a large body of evidence suggests that depression involves dysregulation of GABAergic and glutamatergic transmission across brain, resulting in aberrant stress responsivity, anxiety, and dysphoria [41]. Addressing underlying brain inflammation may be important because inflammation propagates in a feed-forward fashion that has proven very difficult to interrupt in all inflammatory diseases. Furthermore, inflammatory signaling also regulates both GABA and glutamate receptor expression [50] and various brain networks [51], suggesting that short term enhancement of GABA systems will be overcome by the effects of inflammatory signaling unless or until the activated inflammatory pathways are inhibited effectively. Thus, we propose that pleotropic actions of allopregnanolone are essential to its therapeutic potential in PPD and possibly other forms of depression and inflammatory brain disease.

Further investigation of these hypotheses may lead to a better understanding of the etiology of PPD and help clinicians determine what treatment pathways are best for their patients.

## Other neurosteroids under investigation for PPD and beyond

### Zuranolone

Zuranolone, developed by Sage Therapeutics, received FDA approval in August 2023 as the first oral treatment for postpartum depression [52]. Zuranolone is a neuroactive steroid that was developed as an oral agent, and is also a GABA_A_ receptor positive allosteric modulator [35]. It is important to note that zuranolone is not an oral form of brexanolone. It is a novel compound that modulates the activity of both synaptic and extrasynaptic GABA_A_ receptors [35], which differentiates it from benzodiazepines, as the benzodiazepines only target synaptic GABA_A_ receptors [53, 54]. It is also a fast-acting antidepressant with onset of action first noted by day 3 of a 15 day treatment trial. The phase 2 and 3 double blind studies of zuranolone have demonstrated efficacy and safety of zuranolone vs placebo in the outpatient treatment of adult women with PPD [55, 56]. It has also been studied in major depressive disorder (MDD) outside of the postpartum period [57].

There have been two phase 3 trials of zuranolone for PPD and both demonstrated efficacy (defined as HAM-D change from baseline at day 15 of treatment) [55, 56]. Zuranolone 30 mg/day was studied in the first phase 3 PPD study [55] and zuranolone 50 mg/day was studied in the second phase 3 PPD study[56]. In both studies, the drug was well tolerated, and the primary side effects were mild, including somnolence and drowsiness. The FDA approval for PPD comes with a boxed warning about the risk of sedation while driving for up to 12 hours after taking the drug. It will be recommended that patients take the drug at bedtime to minimize the side effect of sedation.

In the MDD phase 3 clinical trials, there have been mixed outcomes. The negative phase 3 trial studied zuranolone at 30 mg and did not meet the primary endpoint (HAM-D) at Day 15 [57]. The more recent positive studies of zuranolone increased the dose to 50 mg and/or shortened the endpoint to day 3, and added concurrent treatment with a standard antidepressant medication [57]. The FDA did not approve zuranolone for MDD in August 2023 at the time it approved the drug for PPD. The FDA issued a complete response letter (CRL) to Sage Therapeutics regarding the MDD indication and will likely require additional studies to be conducted for MDD approval.

### Ganaxolone

Ganaxolone is a 3β-methylated synthetic analog of allopregnanolone and is also being developed for the treatment of PPD. Ganaxolone is also a positive allosteric modulator with effects on both extrasynaptic and synaptic GABA_A_ receptors. However, it does not have affinity for estrogen or progesterone receptors, which is a marked difference [58]. Clinical trials (NCT03460756; NCT03228394) are underway, but published results are not yet available for the PPD studies [59, 60]. In addition, there is a published randomized, double blind, placebo-controlled clinical trial of ganaxolone in a veteran population with posttraumatic stress disorder (PTSD) that did not show differences between active drug versus placebo [61].

## Other neurosteroids in human clinical trials outside of postpartum depression

We briefly mention the human clinical trials using two other neurosteroids (dehydroepiandrosterone and pregnenolone) outside of postpartum depression. Both compounds are available over the counter and have been used in a number of treatment studies for psychiatric disorders.

Dehydroepiandrosterone (DHEA), an adrenal androgen and neurosteroid, was studied in a small randomized, double-blind, placebo-controlled study in midlife patients (men and women) with midlife onset major or minor depression. Six weeks of treatment with DHEA was associated with an improved HAM-D score and the Center for Epidemiologic Studies Depression Scale ratings compared to baseline (*P* < .01) and 6 weeks of placebo (*P* < .01) [62].

Pregnenolone, an endogenous steroid and neurosteroid is a precursor/metabolic intermediate to steroid hormones. Pregnenolone is converted to progesterone and allopregnanolone, but does not alter estrogens, glucocorticoids or mineralcorticoids in human studies (see below). Like allopregnanolone, pregnenolone inhibits proinflammatory signaling through the TLR4 pathway [42], but it has not yet been studied for inhibition of other TLR pathways. Clinically, it has been investigated in multiple different central nervous system (CNS) and psychiatric disorders including pain disorders [63], schizophrenia [64], bipolar depression [65], and alcohol use disorders [66]. The results suggest that pregnenolone also has potential for these disorders and studies are ongoing in various academic centers.

## Conclusion

Neurosteroid therapeutics, particularly positive allosteric modulators of GABA_A_ receptors with anti-inflammatory properties, are poised to become an important new tool in the treatment of postpartum depression and other major depressive disorders. The unique mechanisms of action and rapid onset of action of this class of psychotropic medication show great promise in moving the field forward and increasing our ability to improve treatment outcomes for our patients. As seen with PPD and brexanolone, there are substantial implications across two generations given the importance of mother-infant attachment during the vulnerable postpartum period. The development of neurosteroid therapeutics for treating postpartum depression is an exciting step forward and will hopefully lead to further scientific discovery and innovation in the field.
